# A New Knowledge Characteristics Weighting Method Based on Rough Set and Knowledge Granulation

**DOI:** 10.1155/2018/1838639

**Published:** 2018-05-31

**Authors:** Zhenquan Shi, Shiping Chen

**Affiliations:** ^1^Business School, University of Shanghai for Science and Technology, Shanghai 200093, China; ^2^Nantong University, Nantong, Jiangsu 226017, China

## Abstract

The knowledge characteristics weighting plays an extremely important role in effectively and accurately classifying knowledge. Most of the existing characteristics weighting methods always rely heavily on the experts' a priori knowledge, while rough set weighting method does not rely on experts' a priori knowledge and can meet the need of objectivity. However, the current rough set weighting methods could not obtain a balanced redundant characteristic set. Too much redundancy might cause inaccuracy, and less redundancy might cause ineffectiveness. In this paper, a new method based on rough set and knowledge granulation theories is proposed to ascertain the characteristics weight. Experimental results on several UCI data sets demonstrate that the weighting method can effectively avoid subjective arbitrariness and avoid taking the nonredundant characteristics as redundant characteristics.

## 1. Introduction

In data mining, in order to effectively classify the knowledge, we need to make proper assessment on the knowledge characteristics sets. Therefore, it is very important to compute the weights of characteristics sets. Weights reflect the role of characteristics in the classification process and directly affect the validity and accuracy of the classifier. The common weighting methods include experts scoring method, fuzzy statistics method [1–3], Analytic Hierarchy Process (AHP) method [4–6], and Principal Component Analysis (PCA) method [7, 8]. In these methods, the a priori knowledge must be used.

Rough set theory was firstly proposed by Pawlak in 1982 [9]. It has become an extremely useful tool to handle the imprecision and uncertainty knowledge [9, 10]. Rough set theory can be used to analyze and process the fuzzy or uncertain data without the a priori knowledge [11–17]. Now, the rough set theory has been widely used in pattern recognition [18–20], data mining [21–23], machine learning [24–29], and other fields [30–36].

In recent years, the rough set method has been studied to calculate the characteristics weight. For instance, based on the concepts of characteristics importance, Wang et al. proposed a method to determine the characteristics weights. However, this method did not consider the influence of decision characteristics on conditional characteristics [37]. Cao and Liang combined the characteristics importance of the rough set and the experts' a priori knowledge to determine the characteristics weight [38]. This method achieved the unity of the subjective a priori knowledge with the objective situations, but it ignored the internal difference in the equivalent partitions. Therefore, some nonredundant characteristics would be handled by redundant characteristics. Bao et al. proposed a method ascertaining characteristics weight based on rough set and conditional information entropy. It avoids some nonredundant characteristics to be handled by redundant characteristics. But in this method the characteristics importance obtained by redundant characteristics was higher than that got by nonredundant characteristics [39]. Zhu and Chen constructed the priority queue of characteristics importance to improve Bao's research. They presented a weighting method based on the conditional information entropy and rough set, but that method also involved additional costs [40].

In this paper, a new knowledge characteristics weighting method based on the rough set and knowledge granulation theory is proposed. The accuracy of equivalent partitions in knowledge characteristics is studied and the difference in equivalence classes is analyzed. Experimental results on several UCI data sets confirm our theoretical results. By comparing the numerical results with those of the AHP method, the PCA method, and two rough set based methods, we can draw the conclusion that our new method can effectively avoid taking nonredundant characteristics as redundant characteristics and can improve classification accuracy.

The rest of the paper is structured as follows. Some basic concepts about rough set are briefly introduced in Section 2. In Section 3, a new knowledge characteristics weighting method is proposed and studied. Some experimental results are given in Section 4 to show the effectiveness of the proposed weighting method. Finally, we end this paper with some conclusions in Section 5.

## 2. Basic Concepts

### 2.1. Rough Set

Rough set theory takes knowledge as a partition of the objects domain. The equivalence relations and equivalence classes produced by the equivalence relations are valid information or knowledge about the objects domain. Let *U* denote the universe of objects, which is a nonempty set. *R*⊆*U* × *U* is the equivalence relation on *U*, called the knowledge on the universe *U*. The equivalence relation *R* divides *U* into the disjoint subsets; it is denoted as *U*/*R* or [*U*]_*R*_, representing all the equivalence classes. For the subset *X* of the universe *U*, there are the equivalence classes [*X*]_*R*_. In general, there are two approximation sets—the lower approximation (set) $\underline{R}(X)=\{x|[x{]}_{R}\subseteq X\}$ and the upper approximation (set) $\bar{R}(X)=\{x|[x{]}_{R}\cap X\neq \phi \}$. The lower approximation (set) of the set *X* is also defined as the positive region $POS(X)=\underline{R}(X)$. The set ${BND}_{R}(X)=\bar{R}(X)-\underline{R}(X)$ will be referred to as the *R*-boundary region of *X*. Obviously, when the border area is larger, the set *X* divided by *R* is rougher. Therefore, the roughness of rough set *X* about the equivalence relation *R* can be achieved; it is denoted by

(1) $$\begin{array}{c} D_{R}\left(\;\bar{R}\left(\;X\;\right),\underline{R}\left(\;X\;\right)\;\right)=\frac{\left|BND\left(X\right)\right|}{\left|\bar{R}\left(X\right)\right|}. \end{array}$$

The accuracy of rough set *X* about the equivalence relation *R* is defined as

(2) $$\begin{array}{c} \rho_{R}\left(\;X\;\right)=1-D_{R}\left(\;\bar{R}\left(\;X\;\right),\underline{R}\left(\;X\;\right)\;\right)=\frac{\left|\underline{R}\left(X\right)\right|}{\left|\bar{R}\left(X\right)\right|}, \end{array}$$

where |·| represents the number of the elements in the collection, 0 ≤ *ρ*_*R*_(*X*) ≤ 1. When *ρ*_*R*_(*X*) = 1, *X* is defined as the accuracy set about the equivalence relation *R*. When *ρ*_*R*_(*X*) < 1,  *X* is defined as the rough set about the equivalence relation *R*.

Suppose *P* and *Q* are two equivalence relations about the universe *U*, if *P*⊆*Q*, for ∀*x* ∈ *U*, there is [*x*]_*P*_⊆[*x*]_*Q*_. Thus, the equivalence classes *U*/*P* can be considered finer than the equivalence classes *U*/*Q* and the knowledge (*U*, *P*) is more accurate than the knowledge (*U*, *Q*); see [37–40] for details.

### 2.2. Knowledge Granularity

By the rough set theory, people learn that knowledge is related to the equivalence classes, which shows that knowledge is granular. That is why some scholars also identify the structure of knowledge granularity by the equivalence classes and calculate the size of the knowledge granularity [39].

Suppose that *K* = (*U*, *R*) is a knowledge base, and *R* is an equivalence relation, also known as knowledge. Knowledge granularity is defined as

(3) $$\begin{array}{c} GD\left(\;R\;\right)=\frac{\left|R\right|}{{\left|U\right|}^{2}}. \end{array}$$

If the granularity of *R* reaches its minimum, then GD(*R*) = |*U* | /|*U*|^2^ = 1/|*U*|. If *R* reaches the universe *U*, i.e., the granularity reaches its maximum, then GD(*R*) = |*U*|^2^/|*U*|^2^ = 1. If (*u*, *v*) ∈ *R*, it indicates that the objects *u* and *v* belong to the same equivalence class with the equivalence relation *R*; they are indiscernible. Obviously the smaller GD(*R*) is, the stronger the discernibility of *R* becomes.

Assume that *R* is an equivalence relation, *K* = (*U*, *R*) is a knowledge base, and *U*/*R* = {*X*_1_, *X*_2_,…, *X*_*n*_} is the equivalence class. According to (3), the knowledge granularity can be expressed as

(4) $$\begin{array}{c} GD\left(\;R\;\right)=\overset{n}{\underset{i=1}{\sum }}\frac{{\left|X_{i}\right|}^{2}}{{\left|U\right|}^{2}}. \end{array}$$

And the discernibility of *R* is defined as

(5) $$\begin{array}{c} Dis\left(\;R\;\right)=1-GD\left(\;R\;\right). \end{array}$$

According to (4), there is Dis(*R*) = 1 − ∑_*i*=1_^*n*^(|*X*_*i*_|^2^/|*U*|^2^). Therefore, we have 0 ≤ Dis(*R*) ≤ 1 − 1/|*U*|.

## 3. Knowledge Characteristics Weighting Based on Rough Set and Knowledge Granulation

Cao and Liang calculated the characteristics weights by the cardinality of the positive region set over the cardinality of the discourse set, but the results may be inaccurate [38]. For example, on the field *U* = {1,2, 3,4, 5,6, 7,8, 9}. Let *X* = {1,2, 3,4, 5,8}, and let *R*_1_ and *R*_2_ be defined as the equivalence relation on *U*. Then the following equivalence classes can be obtained:

(6) UR1=1,2,3,4,5,6,7,8,9,UR2=1,2,3,4,5,6,7,8,9.

Their positive areas about *X* on *R*_1_ and *R*_2_ are $\underline{R_{1}}(X)=\underline{R_{2}}(X)=\{1,2,3,4\}$. The weight of the knowledge characteristics *w*_*R*_1__(*X*) = Card(POS_*R*_1__(*X*))/Card(*U*) = 4/9, in which Card(*X*) represents the number of the elements in the collection *X*. And the weight is also shown in *w*_*R*_2__(*X*) = Card(POS_*R*_2__(*X*))/Card(*U*) = 4/9. Thus *w*_*R*_2__(*X*) = *w*_*R*_1__(*X*). It is obvious that the characteristics weights are the same, but the equivalence classes of these two characteristics are different.

In order to solve the problems above, we use the knowledge granularity to study the relationship between the various subsets in the complex sets of the equivalence classes and propose a method based on the knowledge granularity to compute the discernibility of knowledge characteristics. Then, the knowledge characteristics weights according to the relationship between the discernibility and the weights of knowledge characteristics will be determined.

### 3.1. The Discernibility of Knowledge Characteristics

We first give a definition about the discernibility of the knowledge characteristics.

**Definition 1 .**

Suppose that *K* = (*U*, *R*) is a knowledge base, *R* is the equivalence relation, and *r* ∈ *R* is a characteristic. Let *U*/*R* = {*X*_1_, *X*_2_,…, *X*_*n*_} and *U*/(*R* − {*r*}) = {*Y*_1_, *Y*_2_,…, *Y*_*m*_}. Then, the discernibility of *r* is denoted by

(7) $$\begin{array}{c} Dis\left(\;r\;\right)=Dis\left(\;R\;\right)-Dis\left(\;R-\left\{\;r\;\right\}\;\right). \end{array}$$

By Definition 1, we know that the larger Dis(*r*) is, the more discernible the ability of *r* becomes. When we select two objects randomly on *U*, there are |*U*|^2^ ways. After adding characteristic *r* into (*R* − {*r*}), the characteristic discernibility increases from |*R* − {*r*}| to |*R*|. Thus, the number of equivalence classes is more than or equal to the original set. Thus, the ability of such discernibility is improved, and the discernibility increases.

**Theorem 2 .**

Let *r* ∈ *R*,  *U*/*R* = {*X*_1_, *X*_2_,…, *X*_*n*_},  *U*/(*R* − {*r*}) = {*Y*_1_, *Y*_2_,…, *Y*_*m*_}, and denote Dis(*r*) as discernibility of *r*; then there is 0 ≤ Dis(*r*) ≤ 1 − 1/|*U*|.

**Proof**

From (4) and (5), we have

(8) Disr=DisR−DisR−r=1−GDR−1−GDR−r=∑j=1mYj2U2−∑i=1nXi2U2=∑j=1mYj2−∑i=1nXi2U2.

After adding characteristic *r* into (*R* − {*r*}), the characteristic discernibility increases from |*R* − *r*| to |*R*|, and the number of equivalence classes increases. Thus, there exists *Y*_*j*_ ∈ *U*/(*R* − {*r*})  (1 ≤ *j* ≤ *m*) such that *Y*_*j*_ = ⋃_*k*=1_^*n*^*X*_*k*_. And we have

(9) Yj2=⋃k=1nXk2=∑k=1nXk2≥∑k=1nXk2,∑j=1mYj2−∑i=1nXi2≥0,

which shows Dis(*r*) ≥ 0.

When the granularity of [*X*]_*R*_ attains its minimum, there is only one element in *X*_*i*_. When *U*/(*R* − {*r*}) = {*Y*_1_, *Y*_2_,…, *Y*_*m*_} reaches the universe *U*,  Dis(*r*) reaches its maximum. Then we obtain

(10) Disr=∑j=1mYj2−∑i=1nXi2U2=U2−UU2=1−1U.

Thus, 0 ≤ Dis(*r*) ≤ 1 − 1/|*U*| is proved.

### 3.2. Method to Determine Characteristics Weight

To propose our new characteristics weight method, we further give two definitions.

**Definition 3 .**

Suppose that *K* = (*U*, *R*) is a knowledge base and *R* = *C*∩*D*, where *C* denotes the condition characteristics and *D* denotes the decision characteristics. [*X*]_*R*_ = *U*/*D* = {*X*_1_, *X*_2_,…, *X*_*n*_} identifies the equivalence classes on the universe *U* equivalence partitioned by the decision characteristics *D*. Dis(*C*) is the discernibility of *C* on the universe *U*. The discernibility of the knowledge characteristics on [*X*]_*R*_ is defined as

(11) $$\begin{array}{c} KCDis\left(\;C\;\right)=\rho_{c}\left(\;X\;\right)\text{Dis}\left(\;C\;\right). \end{array}$$

According to (2) and (5), we have the following formulation of KCDis(*C*):

(12) KCDisC=ρcX1−GDC=Rc_XRc¯X1−Rc¯X2U2=∑i=1nXi2Rc_Xi/Rc¯Xi1−Rc¯Xi2/U2U.

**Definition 4 .**

Suppose that *K* = (*U*, *R*) is a knowledge base and *R* = *C*∩*D*, where *C* is the condition characteristics and *D* is the decision characteristics. [*X*]_*R*_ = *U*/*D* = {*X*_1_, *X*_2_,…, *X*_*n*_} identifies the equivalence classes on the universe *U* equivalence partitioned by the decision characteristics *D*. For condition characteristics *c* ∈ *C*, the discernibility of *C* is KCDis(*C*) and the discernibility of (*C* − {*c*}) is KCDis(*C* − {*c*}). Then the discernibility of the *c*  (∀*c* ∈ *C*) is defined as

(13) $$\begin{array}{c} \text{KCDis}\left(\;c\;\right)=\text{KCDis}\left(\;C\;\right)-\text{KCDis}\left(\;C-\left\{\;c\;\right\}\;\right). \end{array}$$

According to Definitions 3 and 4, we present a new formula to compute the weight of characteristic in the following definition. Detailed computation process is shown in Algorithm 1.

**Definition 5 .**

Suppose that *K* = (*U*, *R*) is a knowledge base and *R* = *C*∩*D*, where *C* denotes the condition characteristics and *D* denotes the decision characteristics. [*X*]_*R*_ = *U*/*D* = {*X*_1_, *X*_2_,…, *X*_*n*_} identifies the equivalence classes on the universe *U* equivalence partitioned by the decision characteristics *D*. KCDis(*C*) is the discernibility of the knowledge characteristics on [*X*]_*R*_ equivalence partitioned by the condition characteristics *C*. For any conditional characteristics *c* ∈ *C*, the weight of the characteristic is defined as

(14) $$\begin{array}{c} W\left(\;c\;\right)=\frac{\text{KCDis}\left(c\right)}{\sum_{c\in C}\text{KCDis}\left(c\right)}. \end{array}$$

**Theorem 6 .**

Assume that *X* = *U*/*D* = {*X*_1_, *X*_2_,…, *X*_*n*_} is the equivalence class on the universe *U* equivalence partitioned by the characteristics *D*. For any condition characteristics *c* ∈ *C*, KCDis(*c*) is the discernibility of *c* to *U*, and it satisfies 0 ≤ KCDis(*c*) ≤ 1 − 1/|*U*|.

**Proof**

By the rough set theory, we know that

(15) $$\begin{array}{c} 0\leq \rho_{c}\left(\;X\;\right)=\frac{\left|\underline{R_{C}}\left(X\right)\right|}{\left|\bar{R_{C}}\left(X\right)\right|}\leq 1. \end{array}$$

According to Theorem 2, we have 0 ≤ Dis(*C*) ≤ 1 − 1/|*U*|. Thus it is easy to check that 0 ≤ KCDis(*C*) = *ρ*_*C*_(*X*)Dis(*C*) ≤ 1 − 1 | *U*|.

**Theorem 7 .**

Assume that *X* = *U*/*D* = {*X*_1_, *X*_2_,…, *X*_*n*_} is the equivalence class on the universe *U* equivalence partitioned by the decision characteristics *D*. For any condition characteristics *c* ∈ *C*, *KCDis*(*c*) is the discernibility of *c* to *U*. Then

1. if *P* and *Q* are two equivalence relations on *U* and *P*⊆*Q*, then KCDis(*P*) ≤ KCDis(*Q*);
2. 0 ≤ KCDis(*c*) ≤ 1 − 1/|*U*|.

**Proof**

According to Definition 3, there are $Y=\bar{R_{C}}(X)=\{Y_{1},Y_{2},\ldots ,Y_{n}\}$ and $Z=\bar{R_{C-\{c\}}}(X)=\{Z_{1},Z_{2},\ldots ,Z_{m}\}$. According to (11), there is

(16) KCDisc=ρCXDisC−ρC−cXDisC−c=∑i=1nXiRC_Xi/RC¯Xi1−RC¯Xi2/U2−RC−c_Xi/RC−c¯Xi1−RC−c¯Xi2/U2U=∑i=1nXiRC_Xi/RC¯Xi∑j=1n1−Yj2/U2−RC−c_Xi/RC−c¯Xi∑k=1m1−Zk2/U2U.

(1) For the universe *U*, *P* and *Q* are two equivalence relations on the universe *U*. Let *P* = *Q* − {*q*}  (∀*q* ∈ *Q*). There are $Y=\bar{R_{Q}}(X)=\{Y_{1},Y_{2},\ldots ,Y_{n}\}$ and $Z=\bar{R_{Q-\{q\}}}(X)=\{Z_{1},Z_{2},\ldots ,Z_{m}\}$. There exists *Z*_*j*_ ∈ *X*/(*Q* − {*q*})  (1 ≤ *j* ≤ *m*) such that *Z*_*j*_ = ⋃_*k*=1_^*n*^*Y*_*k*_.
For the universe *U*, there are $\underline{R_{Q}}(X)⊇\underline{R_{Q-\{q\}}}(X)=\underline{R_{P}}(X)$ and $\bar{R_{Q}}(X)\subseteq \bar{R_{Q-\{q\}}}(X)=\bar{R_{P}}(X)$. So the following is satisfied:
  (17) $$\begin{array}{c} \rho_{P}\left(\;X\;\right)=\frac{\left|\underline{R_{P}}\left(X\right)\right|}{\left|\bar{R_{P}}\left(X\right)\right|}\leq \rho_{Q}\left(\;X\;\right)=\frac{\left|\underline{R_{Q}}\left(X\right)\right|}{\left|\bar{R_{Q}}\left(X\right)\right|}. \end{array}$$
When *Z*_*j*_ = ⋃_*k*=1_^*n*^*Y*_*k*_, we have |*Z*_*j*_|^2^ = |⋃_*k*=1_^*n*^*Y*_*k*_|^2^ = (∑_*k*=1_^*n*^|*Y*_*k*_|^2^) ≥ ∑_*k*=1_^*n*^|*Y*_*k*_|^2^ and
  (18) $$\begin{array}{c} \overset{n}{\underset{j=1}{\sum }}\left(\;1-\frac{{\left|Y_{j}\right|}^{2}}{{\left|U\right|}^{2}}\;\right)\geq \overset{m}{\underset{k=1}{\sum }}\left(\;1-\frac{{\left|Z_{k}\right|}^{2}}{{\left|U\right|}^{2}}\;\right). \end{array}$$
Substituting (18) into (16), we obtain
  (19) $$\begin{array}{c} \overset{n}{\underset{i=1}{\sum }}\frac{\left|X_{i}\right|}{\left|U\right|}\frac{\left|\underline{R_{Q}}\left(X_{i}\right)\right|}{\left|\bar{R_{Q}}\left(X_{i}\right)\right|}\overset{n}{\underset{j=1}{\sum }}\frac{\left|Y_{j}\right|}{{\left|U\right|}^{2}}\geq \overset{n}{\underset{i=1}{\sum }}\frac{\left|X_{i}\right|}{\left|U\right|}\frac{\left|\underline{R_{P}}\left(X_{i}\right)\right|}{\left|\bar{R_{P}}\left(X_{i}\right)\right|}\overset{m}{\underset{k=1}{\sum }}\frac{\left|Z_{k}\right|}{{\left|U\right|}^{2}}. \end{array}$$
Therefore, KCDis(*P*) ≤ KCDis(*Q*).
(2) From (16) and (19), we have KCDis(*c*) ≥ 0. When *C* becomes the universe *U*, it partitions the universe *U* into equivalence classes (one class comprises individual elements). For this case, KCDis(*c*) reaches its maximum KCDis(*c*) = 1/|*U*|. Therefore, 0 ≤ KCDis(*c*) ≤ 1 − 1/|*U*| is obtained.

## 4. Experimental Results

In this section, some experiments are used to show the effectiveness of our new method. The data used in our experiments come from the Pima Indians Diabetes Data Set, which includes a total of 768 cases, of which 392 are valid, and the rest of the data cases' characteristics values are missing. Note that the Pima Indians Diabetes Data Set is no longer available due to permission restrictions.

In actual computations, we use these 392 cases for experimentation. The condition characteristics information includes “plasma glucose concentration at 2 hours in an oral glucose tolerance test”, “diastolic blood pressure (mm Hg)”, “triceps skin fold thickness (mm)”, “2-hour serum insulin (mu U/ml)”, “body mass index (weight in kg/(height in m)^2^)”. The data set is given in Table 1, where “*c*1”, “*c*2”, “*c*3”, “*c*4”, and “*c*5” denote the condition characteristics, respectively. “*d*” stands for the decision characteristics “class variable (0 or 1)”. Then the condition characteristics values are discretized to different levels as “*A*, *B*, *C*” or “*A*, *B*, *C*, *D*”; see Table 2.

According to Algorithm 1, the following characteristics weights can be obtained:

(20) Wc1=0.3625,Wc2=0.0451,Wc3=0.2388,Wc4=0.2848,Wc5=0.0688.

Two experiments are conducted to show the advantages of our new method. The first experiment is to compare different rough set based methods with our method. The second one is to compare the AHP and PCA methods with our method. Both comparisons can show that our new proposed method is more effective than those methods.

In the first experiment, we also choose two rough set-based methods. One is based on the dependence in rough set theory to calculate the characteristics weight. The other is based on rough sets and conditional information entropy.

In knowledge bases *K* = (*U*, *R*) and *R* = *C*∩*D*, the dependence of the characteristic is defined as *γ*_*C*_(*D*) = |POS_*B*_(*D*)|/|*U*|. The characteristics importance Sig(*c*) = *γ*_*C*_(*D*) − *γ*_*C*−{*c*}_(*D*). Then the characteristics weight is *W*_1_(*c*) = Sig(*c*)/∑_*a*∈*C*_Sig(*a*) [39]. By calculation, we have

(21) W1c1=0.5,W1c2=0,W1c3=0.3,W1c4=0.2,W1c5=0.

In knowledge bases *K* = (*U*, *R*) and *R* = *C*∩*D*, the dependence of the characteristic is defined as *I*(*D*∣*C*). *I*(*D*∣*C*) = ∑_*i*=1_^*m*^(|*C*_*i*_^2^|/|*U*^2^|)∑_*j*=1_^*k*^(|*D*_*j*_∩*C*_*i*_|/|*C*_*i*_|)(1 − |*D*_*j*_∩*C*_*i*_|/|*C*_*i*_|), and the characteristics importance Sig(*c*) = *I*(*D*∣*C* − {*c*}) − *I*(*D*∣*C*). Then the characteristics weight is *W*_2_(*c*) = Sig(*c*)/∑_*a*∈*C*_Sig(*a*) [40]. By calculation, we have

(22) W2c1=0.5,W2c2=0,W2c3=0.2857,W2c4=0.2143,W2c5=0.

**Figure 1:**
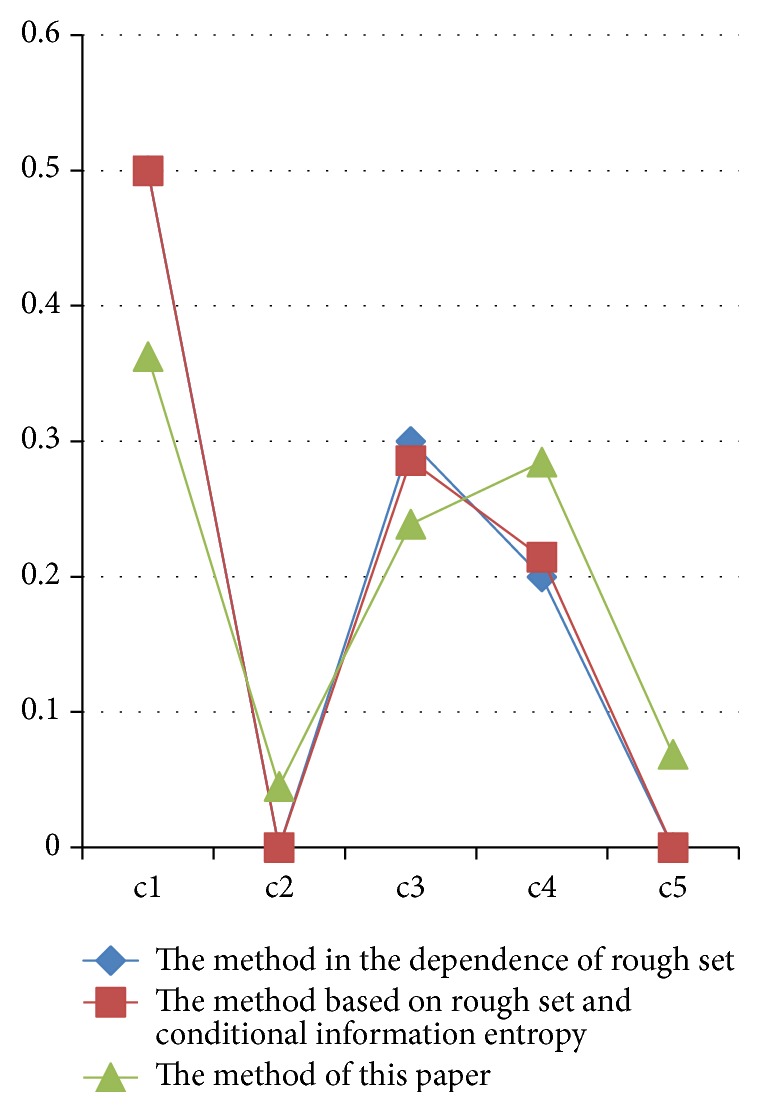
Comparison of three methods based on rough set.

In Table 3, we list the weighting results of the three methods based on rough set.  Figure 1 clearly shows their comparison. From Table 3 and Figure 1, it shows that when the methods based on the dependence of rough set and the method based on the rough set and conditional information entropy are used to calculate the characteristics weights, the weights of “*c*2” and “*c*5” are redundant. But when the proposed method is used to calculate the characteristics weights, the results were not redundant. There is a little relation between “diastolic blood pressure (mm Hg)”, “body mass index (weight in kg/(height in m)^2^)”, and diabetes, but they are related. So, from this point of view, the new method is more accurate than the other two rough set-based methods.

In the second experiment, the AHP method and the PCA method are used to calculate the characteristics weight. We also compare their results with ours.

For the AHP method, we construct the analytic hierarchy matrix according to the opinion of medical experts [41]. Then we obtain the weights:

(23) W3c1=0.0604,W3c2=0.1012,W3c3=0.3103,W3c4=0.1815,W3c5=0.3465.

For the PCA method, we select the representative variables through the transformation of multiple variables. Then the SPSS software is used to seek the explanation of the total variance and component of the matrix. We take principal components variance contribution rate as weight [41] and finally normalize them to get the weights:

(24) W4c1=0.432,W4c2=0.1114,W4c3=0.0978,W4c4=0.2568,W4c5=0.1019.

**Figure 2:**
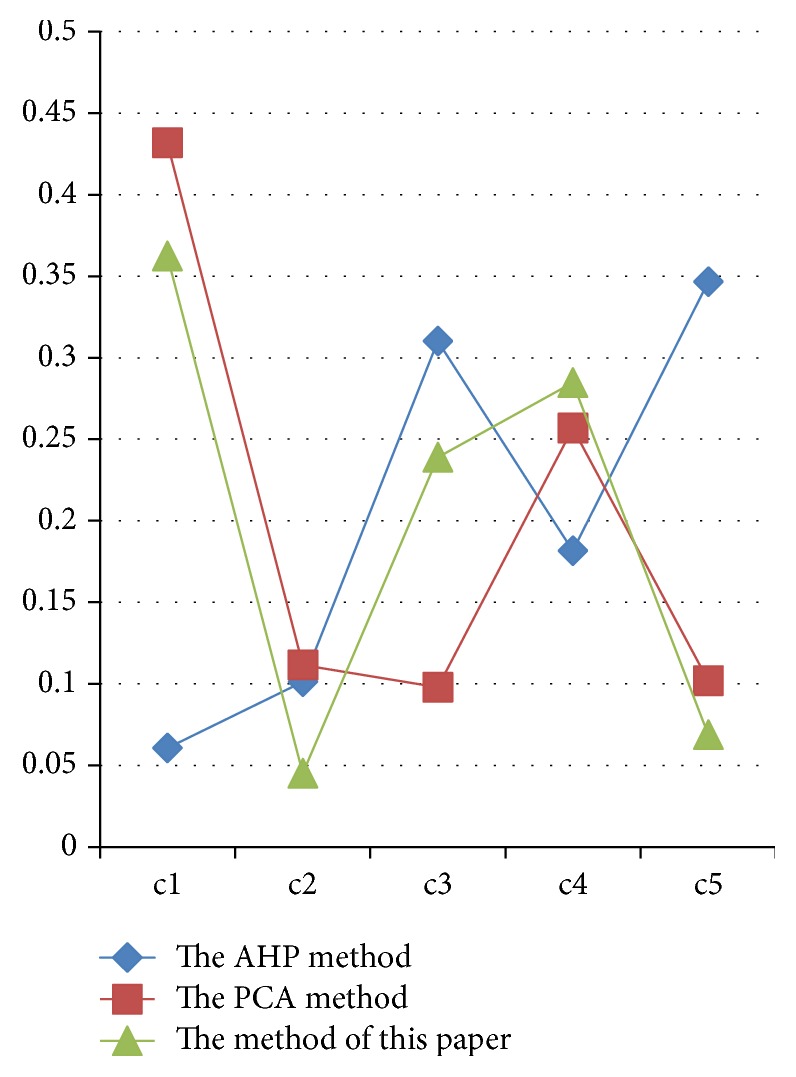
Comparison of three methods.

The weighting results are given in Table 4.  Figure 2 shows the comparison between the proposed method and two well-known methods. From Table 4 and Figure 2, it is easy to check that the rank of the results calculated with our method is “*c*1” > “*c*4” > “*c*3” > “*c*5” > “*c*2”. It shows that there is a closed relation between “plasma glucose concentration at 2 hours in an oral glucose tolerance test” and diabetes, and there is a little relation between “diastolic blood pressure (mm Hg)” and diabetes. These results are synthetic optimization on the results calculated by AHP and PCA from Figure 2. By consulting the medical experts, the results calculated by our method are more accordant with the actual situation.

However, the Analytical Hierarchy Process (AHP) method is based on the subjective judgment of the experts and the Principal Component Analysis (PCA) method needs to extract representative principal components and increase an additional a priori information and evaluation criteria. Therefore, these two methods cannot objectively reflect the weight distribution. The new method does not need the prior knowledge, but the obtained weights are in line with the actual situation.

From the above discussion, the weighting method based on rough set can avoid the arbitrariness of subjective judgment. In addition, the weighting method with granularity theory can effectively avoid taking nonredundant characteristics as redundant characteristics. We can conclude that our new method reasonably distributes the weight for each characteristic. The weights basically reflect the importance of each characteristic and can also objectively reflect the actual situation of the patient's body. Thus, the proposed method is a powerful method in knowledge classification.

## 5. Conclusions

Knowledge characteristics can help us have a good understanding of the knowledge base. The determination of knowledge characteristics weight can help us effectively classify the knowledge base, so as to achieve the purpose of knowledge management and decision making. In this paper, based on rough set theory and knowledge granularity theory, the weights of knowledge characteristics are determined. Experimental results show that the proposed method can effectively avoid taking nonredundant characteristics as redundant characteristics and can effectively determine the weights of knowledge characteristics.

## Figures and Tables

**Algorithm 1:**
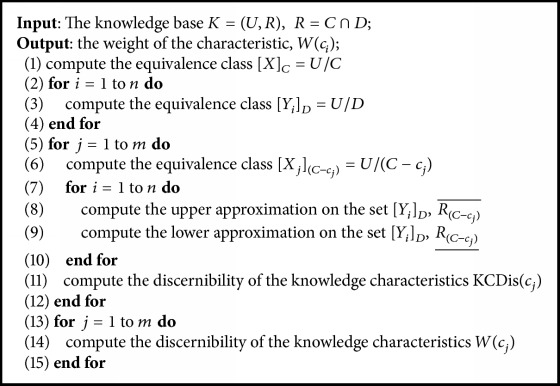
Method to determine characteristics weight.

**Table 1:** The Pima Indians Diabetes Data Set.

*U*	*c*1	*c*2	*c*3	*c*4	*c*5	*d*
*U*1	89	66	23	94	28.1	0
*U*2	137	40	35	168	43.1	1
*U*3	78	50	32	88	31	1
*U*4	197	70	45	543	30.5	1
*U*5	189	60	23	846	30.1	1
*U*6	166	72	19	175	25.8	1
*U*7	118	84	47	230	45.8	1
*U*8	103	30	38	83	43.3	0
*U*9	115	70	30	96	34.6	1
*U*10	126	88	41	235	39.3	0
⋮	⋮	⋮	⋮	⋮	⋮	⋮
*U*384	100	84	33	105	30	0
*U*385	81	74	41	57	46.3	0
*U*386	187	70	22	200	36.4	1
*U*387	121	78	39	74	39	0
*U*388	181	88	44	510	43.3	1
*U*389	128	88	39	110	36.5	1
*U*390	88	58	26	16	28.4	0
*U*391	101	76	48	180	32.9	0
*U*392	121	72	23	112	26.2	0

**Table 2:** The discretized Pima Indians Diabetes Data Set.

*U*	*c*1	*c*2	*c*3	*c*4	*c*5	*d*
*U*1	*A*	*B*	*B*	*B*	*A*	0
*U*2	*C*	*A*	*C*	*C*	*C*	1
*U*3	*A*	*A*	*C*	*B*	*B*	0
*U*4	*D*	*B*	*D*	*D*	*B*	1
*U*5	*D*	*B*	*B*	*D*	*B*	1
*U*6	*D*	*B*	*A*	*C*	*A*	0
*U*7	*B*	*A*	*D*	*C*	*C*	0
*U*8	*B*	*B*	*C*	*B*	*C*	1
*U*9	*B*	*B*	*C*	*B*	*B*	0
*U*10	*C*	*C*	*D*	*C*	*B*	0
⋮	⋮	⋮	⋮	⋮	⋮	⋮
*U*384	*D*	*B*	*C*	*B*	*B*	0
*U*385	*A*	*B*	*D*	*A*	*C*	1
*U*386	*D*	*B*	*B*	*C*	*B*	0
*U*387	*C*	*B*	*C*	*B*	*B*	0
*U*388	*D*	*B*	*D*	*D*	*C*	0
*U*389	*C*	*B*	*C*	*B*	*B*	0
*U*390	*A*	*A*	*B*	*A*	*A*	1
*U*391	*B*	*B*	*D*	*C*	*B*	1
*U*392	*C*	*B*	*B*	*B*	*A*	0

**Table 3:** Comparison of three methods based on rough set.

Method	*c*1	*c*2	*c*3	*c*4	*c*5
The method based on the dependence of rough set	0.5000	0	0.3000	0.2000	0
The method based on rough set and conditional information entropy	0.5000	0	0.2857	0.2143	0
The method of this paper	0.3625	0.0451	0.2388	0.2848	0.0688

**Table 4:** Comparison of different types of methods.

Method	*c*1	*c*2	*c*3	*c*4	*c*5
The AHP method	0.0604	0.1012	0.3103	0.1815	0.3465
The PCA method	0.432	0.1114	0.0978	0.2568	0.1019
The method of this paper	0.3625	0.0451	0.2388	0.2848	0.0688

## Data Availability

The data used to support the findings of this study are available from the corresponding author upon request.
